# Barriers to kidney transplant referral in the state of Minas Gerais: nephrologists’ perspectives

**DOI:** 10.1590/2175-8239-JBN-2025-0215en

**Published:** 2026-08-10

**Authors:** Cristianne de Medeiros Nogueira, Luiza Cunha Martins, Helady Sanders-Pinheiro

**Affiliations:** 1 Universidade Federal de Juiz de Fora, Núcleo Interdisciplinar de Estudos e Pesquisas em Nefrologia, Juiz de Fora, MG, Brazil.; 2 Hospital Regional Dr. João Penido, Serviço de Nefrologia, Juiz de Fora, MG, Brazil.; 3 Universidade Federal de Juiz de Fora, Hospital Universitário, Serviço de Transplante Renal, Juiz de Fora, MG, Brazil.

**Keywords:** Kidney Transplantation, Barriers to Access of Health Services, Life Support Systems, Nephrologists, Health Personnel, Referral and Consultation, Waiting Lists

## Abstract

**Background:**

In Brazil, there is a low percentage of patients on the waiting list for kidney transplantation (KT), even within a public health system with full coverage. Among the factors influencing referral, those related to the nephrologist are the least studied. We aimed to investigate the barriers to KT referral from the perspective of nephrologists in the state of Minas Gerais.

**Methods:**

Cross-sectional study of a convenience sample of active nephrologists. We collected data through an electronic survey, including demographic profile, education, professional profile, knowledge about indications (6) and contraindications (12), and clinical practice. Non-parametric analyses were performed.

**Results:**

The 77 nephrologists had a median age of 44.9 years (IQR 39–52) and a median of 12.5 years (IQR 3–23) of clinical practice; all had completed postgraduate training. The median number of expected responses regarding contraindications was 8 (IQR 6–9) out of 12, and 66.2% had more than 50% of correct responses. There were no significant associations with this pattern of responses (< 8). The nephrologists reported knowing the referral process and identified the presence of contraindications as the main cause for non-referral. Centers were public (54.5%) and non-academic (55.8%); 69.2% were located within < 100 km of a KT center, most offered KT as a treatment modality (80.4%), only 14.7% reported difficulties with pre-KT laboratory tests, and 21–40% of patients were reported to be listed (31.2%).

**Conclusion:**

We found knowledge gaps in KT contraindications and risk perception as causes for non-referral. Barriers related to centers and the health system were not reported. These findings support educational initiatives and greater integration between nephrology services and KT centers to enable referrals.

## Introduction

Chronic kidney disease (CKD) affects approximately 10% of the global population, with incidence increasing due to population aging and more frequent diagnosis^1^. As CKD progresses to end-stage renal disease, renal replacement therapies (RRTs)—which include hemodialysis, peritoneal dialysis, and kidney transplantation (KT)—become necessary^2^. In Brazil, the prevalence of patients undergoing RRT continues to rise. The Brazilian Dialysis Census estimates that approximately 157,357 patients were on dialysis in 2023^3^. Compared to dialysis therapies, KT provides better quality of life and overall survival^4,5^. In Brazil, the population undergoing dialysis increased by 56.7% over the past decade (2013–2023); however, the number of KTs performed increased by only 10.6% during the same period^3,6^. The growth in the number of KTs in Brazil has slowed in recent years: after a 42% increase between 2008 and 2012, an increase of only 9% was noted over the past six years, which may have been influenced in part by the COVID-19 pandemic^7^.

This stagnation results from complex barriers, including limitations in the health system, logistical challenges, patient-related factors, and issues related to the healthcare team. The most cited factors include underreporting of brain death, high rates of family refusal, difficulties in maintaining potential donors, a decline in living donors, and a low proportion of patients registered on the waiting list^8,9^.

Although Brazil is globally recognized for its transplant program, within the Unified Health System (*Sistema Único de Saúde*, SUS), which provides full and free coverage, significant disparities remain. In 2024, 6,297 KTs were performed nationwide, meeting only approximately 17% of the estimated demand, while 36,985 patients remained on the waiting list^7^. The public system covers all stages—pre-transplant evaluation, surgery, postoperative follow-up, and immunosuppression—yet the number of KTs performed remains insufficient to meet the population’s needs^7^.

An underestimated cause of this gap is access to the KT waiting list. Eligibility for inclusion requires a reduced glomerular filtration rate (GFR) (< 10–15 mL/min/1.73 m^2^) and the absence of clinical contraindications after evaluation at a specialized transplant center. However, the first step requires referral by a nephrologist to a transplant center^8,9^. The limited available data indicate that only 17.3% to 33.7% of patients are referred, and fewer than half of these proceed to the laboratory evaluation phase^10,11^. This process is primarily influenced by the KT candidate’s clinical condition. Regardless of the patient’s health status, the process may also be affected by patient-related factors (age, education level, sex, and socioeconomic status), healthcare clinical practices (lack of referral, limited knowledge of indications, and communication failures), and system-level barriers (distance from transplant centers and workforce shortages)^11,12^. This critical and early step in accessing KT remains underexplored, particularly regarding factors beyond patient characteristics^9,12,13,14^.

The few national studies addressing barriers to transplantation have mostly focused on the percentage of patients on the waiting list and their subsequent access to KT, since this information is more readily available^15,16,17,18^. Based on data from the Brazilian Transplant Registry and the Brazilian Dialysis Census from 2019 to 2023, the percentage of patients registered on the waiting list ranged from 18% to 20.9%, representing a proportional increase of 34.5% over this period^3,6,19,20,21,22^. In the state of Minas Gerais, from 2016 to 2019, data from the National Transplant System (*Sistema Nacional de Transplantes* – SNT) and the SUS showed that 14.8% of patients were listed, with wide variation across macro regions, from 1.0% in Jequitinhonha to 21.4% in the northern region^23^. However, only two studies have investigated specific aspects related to referral for KT^24,25^. These studies were conducted in regions with markedly different socioeconomic realities, one in the South^25^ and the other in the Northeast^24^ of Brazil. Both explored, from the patients’ perspective, the reasons for not being listed or for not undergoing pre-transplant evaluation. The most frequently reported reasons, even among patients clinically eligible for referral^24^, were lack of information, patient refusal, and difficulties in completing the pre-transplant evaluation^24,25^. These findings highlight that some barriers could be addressed by implementing simple interventions at the levels of dialysis centers and healthcare professionals.

No Brazilian studies have investigated barriers to referral from the perspective of nephrologists, who play a key role at the beginning of the transplant process^14,26^. Minas Gerais is the second Brazilian state in the absolute number of KTs and ranks sixth when adjusted for population size^7^. In 2024, 60% of the estimated demand for transplantation was met across 21 active centers in the state; 3,737 patients were on the waiting list, representing 10.1% of the national total^7^. Compared to the national average, the state has a higher density of nephrologists^27^. Understanding the factors that influence physicians’ decisions and behaviors regarding patient referral for KT evaluation is essential for developing strategies to improve access and reduce disparities^9,26^.

This study aims to investigate the barriers to referral for KT evaluation in Minas Gerais at the levels of healthcare providers, dialysis centers, and the health system, from the perspective of nephrologists, thereby contributing to the development of more effective and context-sensitive planning.

## Methods

### Study Design and Sample

This is a cross-sectional study comprising a convenience sample of nephrologists residing in the state of Minas Gerais who were actively involved in the management of patients with CKD, either receiving conservative treatment or undergoing dialysis. Physicians’ contacts were identified through the Brazilian Society of Nephrology’s registry and participant lists from events organized by the Minas Gerais Society of Nephrology up to 2023. Between December 2023 and June 2024, the lead researcher sent invitations to participate via phone and/or email. Each physician was sent a virtual questionnaire containing the study questions. Up to three invitations were sent to each contact. Nephrologists were included in the study after agreeing to participate and signing the informed consent form (ICF), which was embedded in the electronic questionnaire. The eight sections of the questionnaire were not mandatory and could therefore be viewed upon opening the survey. For questions regarding indications for KT, participants responded to each item using a multiple-choice Likert scale (Strongly agree, Agree, Neither agree nor disagree, Disagree, Strongly disagree). For contraindications, participants were asked to provide an open-ended response indicating their position, selecting one of the following options: absolute contraindication, relative contraindication, or do not know. A pilot study was initially conducted with five participants, during which minor comprehension issues were identified and subsequently corrected. This approach was chosen to enable a broader exploration of the underlying concepts^28,29^. The inclusion criteria were being a practicing nephrologist in Minas Gerais and being involved in the care of patients with CKD, either under conservative management or undergoing dialysis. Questionnaires that were more than 20% incomplete were excluded.

The study was approved by the Human Research Ethics Committee of the Federal University of Juiz de Fora (CAAE 69328523.9.0000.5147; Opinion No. 6.090.544). Participation was only possible after reading and accepting the ICF by clicking on the appropriate field in the electronic form, which preceded the questionnaire.

### Variables

Variables related to factors influencing referral for KT evaluation were collected and grouped into three main categories, namely characteristics of nephrologists, dialysis centers, and the healthcare system^12,13^.

At the nephrologist level, we collected data on the following variables^12,13,30,31^: demographic characteristics — age, sex, and race (White or non-White); professional training — time since graduation (years), time since postgraduate education (years), and type of training (medical residency, specialization only, or both); and professional activity — type of service provided in relation to CKD treatment (dialysis or conservative management) and the employment relationship with the dialysis center (service provider, public servant, owner, or formally employed under labor contract). Knowledge on KT^28,29^ included the following: indications for KT (irreversible kidney injury, GFR of < 10 mL/min/1.73 m^2^ for patients without diabetes or < 15 mL/min/1.73 m^2^ for diabetic patients, patients < 18 years of age, cases with potential living donors, and clinical and surgical eligibility); absolute contraindications (six items) (malignant neoplasms, organ failure without potential treatment, degenerative neurological diseases, severe vasculopathy including iliac vessels, poorly controlled psychiatric disorders, and illicit substance use); and relative or temporary contraindications (six items) (recent immunologic graft loss, acute infection, active peptic ulcer disease, active systemic diseases, recent stroke or acute myocardial infarction (AMI), untreated coronary disease. Reported reasons for non-referral included the presence of contraindications, perception of risks outweighing benefits, and lack of donors.

At the level of the dialysis center, we collected^11,12,13,32,33,34^ the following variables: type of institution (academic or non-academic) and economic nature (public or private); center practices, including whether KT was offered as an RRT option (yes/no), knowledge of referral process (yes/no), number of nephrologists at the center, percentage of patients on the transplant waiting list (< 20%, 20%–40%, > 40%, or unknown), and the professional responsible for referral (physicians, nurses, social workers, psychologists, or no designated professional).

At the level of the health system, data on distance to the transplant center (< 10 km, 10–100 km, or > 100 km) and perceived difficulty in completing pre-transplant tests were collected^12,13,35^.

### Statistical Analysis

Descriptive analysis was performed for all variables, reported as means with standard deviations or medians with interquartile ranges (IQRs), depending on the nature of the data. Normality was assessed using the Kolmogorov–Smirnov test. To evaluate factors associated with lower knowledge regarding contraindications to KT, the sample was divided into two groups: participants with fewer than eight expected responses (< 8 group) and those with eight or more (≥ 8 group), based on the median number of expected responses among the twelve items described above. For comparisons of continuous variables, Student’s t-test or the Mann–Whitney U test was used, according to data distribution. Categorical variables were compared using the chi-square test or Fisher’s exact test, as appropriate. A significance level of p < 0.05 was adopted. All analyses were performed using jamovi (version 2.6), an open-source statistical software (The jamovi project, 2025, https://www.jamovi.org).

## Results

**Figure 1:**
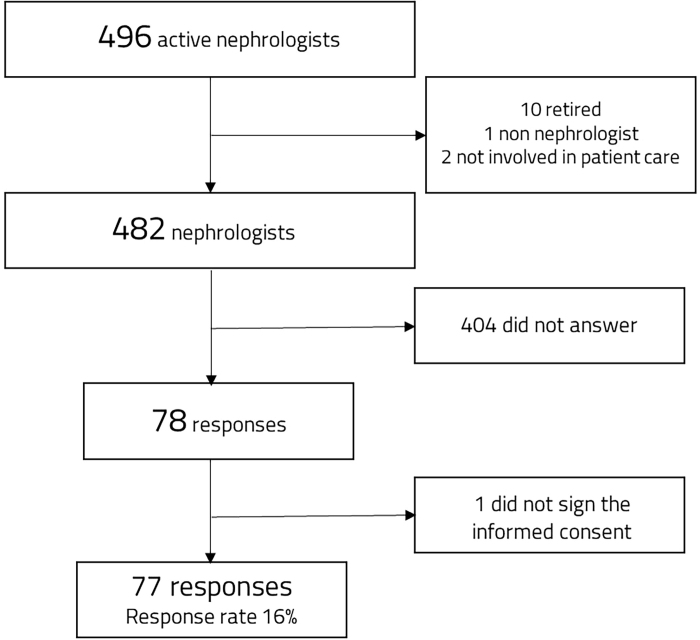
Sample flow.

Of the 496 nephrologists identified, 13 were excluded: 10 were retired, two were not involved in patient care, and one was not a nephrologist. We received 78 responses from the remaining 482 nephrologists, of whom 77 provided the ICF, resulting in a response rate of 16%. No participants were excluded due to incomplete responses (Figure 1). The median age was 44.9 years (IQR, 39–52). Most participants (54.5%) were women and 76.6% identified themselves as White. The median time since graduation was 18 years (IQR, 13–28), the time since completion of postgraduate training was 13 years (IQR, 6–23), and participants had been practicing nephrology for a median of 12.5 years (IQR, 3–23). All participants had completed postgraduate training, most commonly through a medical residency program (79.2%); 93.5% of participants were trained at a transplant center. All participants reported working in a unit that offers both dialysis and conservative CKD management. Regarding employment status, 68.5% were service providers, and only 6.8% were owners of the centers (Table 1).

**Table 1:** Demographic, educational, and practice-related characteristics of nephrologists working in the state of Minas Gerais and of the nephrology centers at which they practice, according to the median number of expected responses regarding knowledge of KT contraindications

Variable	Total (N = 77)	Expected responses regarding KT contraindications		
		< 8 Group (n = 57)	≥ 8 Group (n = 20)	p value
Nephrologist				
Age, years	44.9 (39–52)	44.0 (30–64)	42.5 (31–60)	0.42
Women, n (%)	42 (54.5)	32 (56.1)	10 (50.0)	0.63
Race, n (%)				0.68
*White*	59 (76.6)	43 (75.4)	16 (80.0)	
*Non-White*	18 (23.4)	14 (24.6)	04 (20.0)	
Time since graduation, years	18.0 (13–28)	18.0 (5.0–39.0)	16.0 (6.0–31.0)	0.25
Time since postgraduate training, years	13.0 (6.0–2.03)	13.0 (0–3.07)	12.0 (1.0–2.09)	0.36
Type of postgraduate training, n (%)	18 (1.03–28.0)			0.59
*Medical residency*	61 (79.2)	46 (80.7)	15 (75.0)	
*Specialization*	16 (20.8)	11 (19.3)	05 (25.0)	
Postgraduate training at a KT center	72 (93.5)	53 (93.0)	19 (95.0)	0.75
Experience as a nephrologist, years	12.5 (3–23)	13.0 (1.0–36.0)	12.0 (1.0–31.0)	0.38
Employment relationship, n (%)*				0.09
*Service provider*	59 (68.5)	41 (75.9)	9 (47.4)	
*Public servant*	14 (19.2)	7 (12.9)	7 (36.8)	
*Owner*	5 (6.8)	3 (5.5)	02 (10.5)	
*Formally employed under labor contract*	4 (5.5)	3 (5.5)	01 (5.3)	
Dialysis centers and health system				
Type of institutional funding, n (%)				0.44
*Public*	42 (54.5)	30 (52.6)	12 (60.0)	
*Private*	32 (41.6)	27 (47.4)	8 (40.0)	
Academic institution, n (%)	34 (44.2)	24 (42.1)	10 (50.0)	0.53
Number of nephrologists	6 (4–10)	6.0 (3.0–20.0)	8.0 (1.0–42.0)	0.17
Offers KT as RRT, n (%)	62 (80.4)	47 (82.4)	18 (90.0)	0.42
Distance to a transplant center**				0.76
*Up to 10 km*	44 (58.6)	32 (57.1)	12/20 (60.0)	
*10–100 km*	08 (10.6)	5 (8.9)	3 (15.0)	
*>100 km*	23 (30.6)	18 (32.1)	5 (25.0)	
Familiar with the referral process, n (%)	64 (82.9)	45 (78.9)	18 (90.0)	0.59
% of patients on the waiting list, n (%)				0.36
*< 20%*	14 (18.2)	10 (22.8)	04 (20.0)	
*20%–40%*	24 (31.2)	15 (26.3)	09 (45.0)	
*> 40%*	20 (26)	17 (29.8)	03 (15.0)	
*Does not know*	19 (24.7)	15 (26.3)	04 (20.0)	

Abbreviations – IQR: interquartile range; KT: kidney transplantation; n: number; RRT: renal replacement therapy. Notes – Data are expressed as absolute numbers and percentages or as medians and interquartile ranges (IQR). Between-group comparisons based on the number of expected responses regarding KT contraindications were performed using the Mann–Whitney U test, chi-square test, or Fisher’s exact test.

*Four missing values, three in the ≤ 8 responses group and one in the > 8 responses group.

**One missing value in the < 8 responses group.

**Figure 2:**
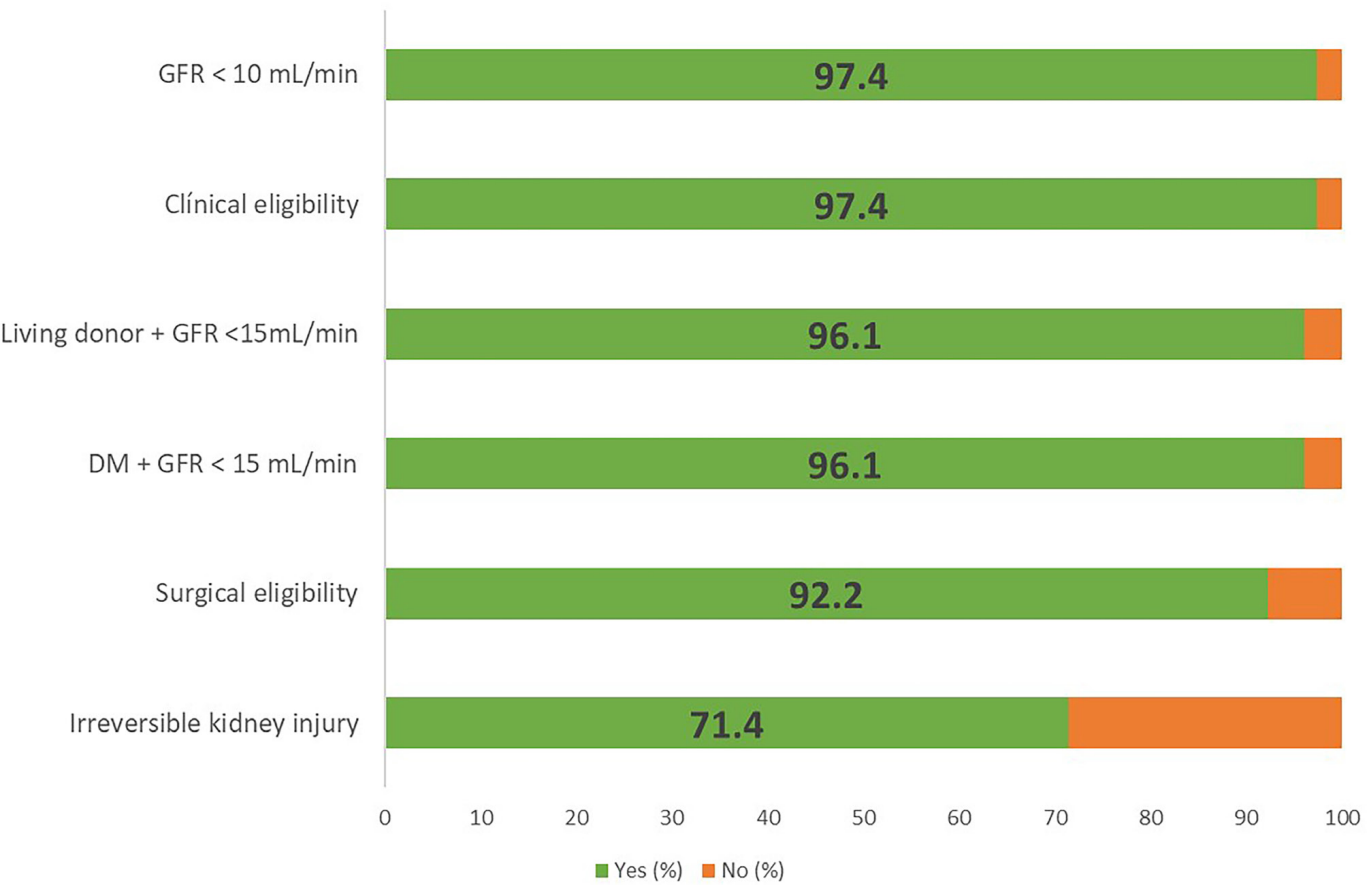
Frequency of expected responses regarding indications for KT.

**Figure 3:**
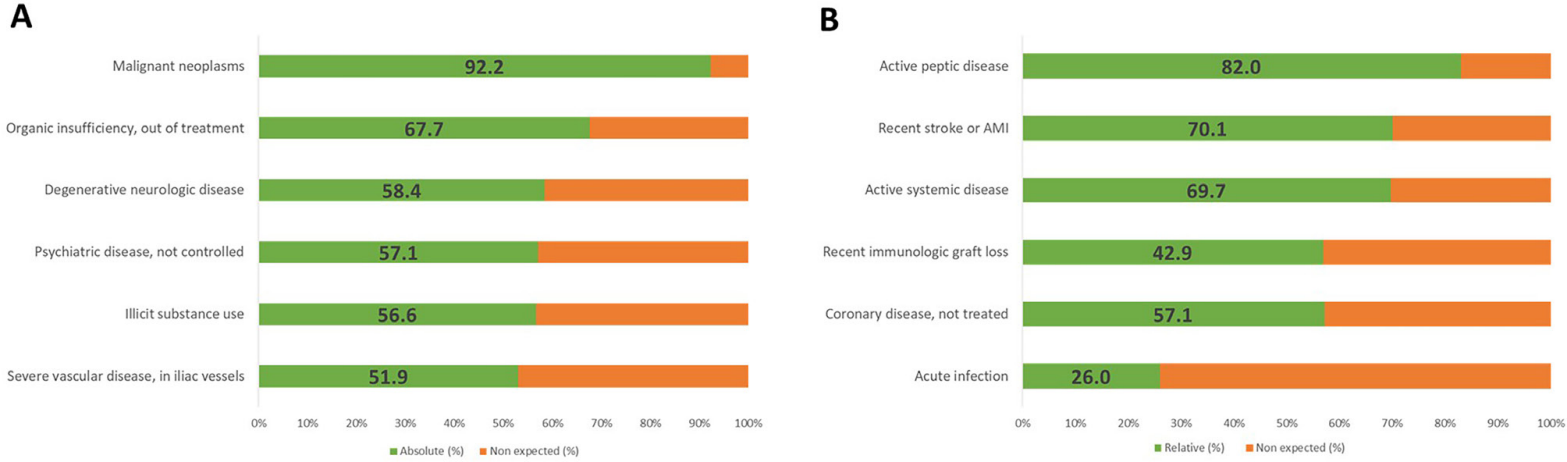
Frequency of expected responses regarding contraindications for KT.

Concerning knowledge of referral criteria, performance on KT indications was very good. More than 90% of participants gave appropriate responses to 4 of the 6 items assessed: clinical eligibility (97.4%), surgical eligibility (92.2%), GFR of < 10 mL/min/1.73 m^2^ (97.4%) for patients without diabetes, and GFR of < 15 mL/min/1.73 m^2^ in patients with diabetes (96.1%). Only 71.4% of participants agreed with the criterion of irreversible kidney injury (Figure 2). An important proportion of unexpected responses was observed regarding the classification of absolute contraindications, and an even higher proportion for relative contraindications (Figure 3A and B). The median number of expected responses for the six absolute contraindications was 4.0 (3–5), and the median for the six relative contraindications was also 4.0 (3-4). The median number of expected responses considering all 12 contraindications was 8.0 (6–9). When the sample was assessed according to the majority (≥ 50%) of expected responses, 66.2% of participants (51/77) correctly identified more than half of contraindications for KT. No significant associations were found between performance in relation to knowledge of contraindications when participants were divided by the number of expected responses above or below the median score (≥ 8 or < 8). The most commonly reported reasons for non-referral were the presence of contraindications (90.8%) and the perception that the risks outweighed the benefits (43.4%); difficulty in accessing transplant centers or completing pre-transplant testing was cited less frequently (21%; Table 1).

Abbreviations – DM, diabetes mellitus; GFR, glomerular filtration rate; KT, kidney transplantation.

Abbreviations – A, absolute; B, relative or temporary; AMI, acute myocardial infarction; KT, kidney transplantation; TIA, transient ischemic attack.

Of the nephrology centers, 54.5% were public, and most (55.8%) were non-academic institutions. The median number of nephrologists per center was 6 (IQR, 4–10). Over 80% reported routinely offering KT as an RRT modality. A quarter of participants (24.7%) did not know the percentage of patients at their center who were registered on the kidney transplant waiting list, and 31.2% reported that 20% to 40% of patients were enlisted (Table 1).

For factors related to the healthcare system, 58.6% of centers were located within 10 km of a transplant center (i.e., likely in the same city), 10.6% were between 10 and 100 km away, and 30.6% were more than 100 km away. Among the nephrologists, 82.9% stated that they were familiar with the procedures involved in the referral process; participants indicated that referrals were performed by physicians and social workers in 90.9% of cases. Only 17.4% of nephrologists reported facing difficulties in performing the pre-transplant laboratory evaluation (Table 1).

## Discussion

This study explored the barriers to referral for KT evaluation from the perspective of nephrologists in the state of Minas Gerais, providing an assessment of how these factors may limit access to the waiting list. The results show that, even among a population of professionals trained in transplant centers and actively involved in dialysis or pre-dialysis care, challenges related to knowledge of KT, particularly regarding contraindications, persist. However, no difficulties were reported at the dialysis center or healthcare system levels. This is the first Brazilian study—and one of the few internationally—to explore factors that influence the early phases of the KT waiting list process, namely referral and laboratory evaluation^11,12,13,30,31,33,34,35^.

The sample consisted of experienced professionals, as indicated by their age and number of years of nephrology practice, with solid postgraduate training at transplant centers; more than 40% of these professionals worked in academic institutions. The average age and sex distribution reflect the profile of nephrology specialists in Brazil^27^. Unfortunately, comparison with nephrologists practicing specifically in the state of Minas Gerais was not possible due to a lack of available data. Conversely, this professional profile contrasts with those previously reported as showing a higher risk of non-referral. Tandon et al.^31^ reported that having more than 10 years of postgraduate training and lacking an academic affiliation were associated with non-referral. Another relevant characteristic is that most participants worked in public, not-for-profit services but were service providers rather than being service owners. These factors have been associated with fewer barriers to referral^11,33,34^.

Despite this specialized profile, the scores assigned to contraindication-related questions indicated a need for improvement. At least one-third of participants showed limited knowledge of KT contraindications, an essential aspect of the referral process. These findings suggest heterogeneity in practical knowledge regarding the criteria for contraindications, which may influence referral decisions. International studies have shown that uncertainty or misconceptions among healthcare professionals represent significant barriers to referral^12,13^. However, the impact of knowledge gaps has been reported in specific subgroups, for example, among Black patients^30^ and in contexts where communication failures between physicians and patients are perceived^36^. To date, no studies have directly assessed knowledge regarding KT indications and contraindications. A potential explanation for the performance observed in our study may lie in the fact that, although international reference guidelines are available^28^, evidence supporting some indications and contraindications is not yet robust and remains an evolving area. Moreover, the national consensus, a timely and essential initiative of the Brazilian Society of Nephrology, was only recently published^37^. Unfortunately, based on the variables collected in our study, we were unable to identify any demographic or educational characteristics associated with low performance, defined by scores below the median of expected responses. This finding suggests that the knowledge gaps related to contraindication criteria for KT are relatively widespread and homogeneous within the sample, regardless of age, number of years since graduation, or type of professional affiliation. Such gaps may reflect more complex challenges relating to applied knowledge, day-to-day clinical practice, and the organization of services.

Based on the evaluated center practices—such as offering KT as an RRT modality, awareness of the referral process, and having designated professionals for referral—we identified no structural barriers to referral. Barriers to referral have been described in dialysis centers experiencing workforce shortages^13,32^ and in for-profit units^11,33,34^. Similarly, we did not identify obstacles related to the healthcare system: nearly 70% of participants reported having access to a KT center within 100 km, which corresponds to a potential travel time of up to three hours, a factor previously described as a barrier to referral^35^. Furthermore, fewer than 20% of participants reported difficulty accessing laboratory evaluation. These findings suggest that, in general, the provisions for access to the Brazilian Unified Health System (*Sistema Único de Saúde —* SUS) are being met for these patients.

The clinical implications of this study are significant. Our findings suggest that improving access to KT requires interventions directed not only at patients but also toward continuing education of healthcare professionals. Targeted educational programs, standardization of clinical protocols, and reinforcement of multidisciplinary collaboration are strategies that may enhance referral practices. Given the considerable regional disparities in KT access and delivery across Brazil, assessing the specific barriers in each region could provide a valuable basis for guiding national strategies. In this context, the present study served as the basis for a nationwide study with the same objectives; however, its results have not yet been published (personal communication, coordinators of the *Encaminha Brasil* Study, July 6, 2025). Another relevant strategy would be the development of national guidelines for KT referral, providing a specific and easily accessible source of information to support professional training and serve as a practical reference for clinicians. Fortunately, as cited above, the Consensus Statement from the Brazilian Society of Nephrology on Referral for KT was recently published^37^.

This study had some limitations. The sample was convenience-based and limited to nephrologists in the state of Minas Gerais, which may restrict the generalizability of the findings to other regions or healthcare contexts in Brazil. Moreover, the representativeness of the sample in relation to the population of nephrologists in Minas Gerais cannot be rigorously assessed due to the lack of demographic data for this professional group. An important issue is the absence of well-established evidence on what should be considered clear indications and contraindications for KT^28^, which may have influenced the criteria we used to define knowledge levels in this study. However, for the purposes of this research, it was methodologically necessary to establish an operational definition of what would be considered an indication or a contraindication for KT. Furthermore, we did not investigate aspects related to continuing education or recent specific specialized training among professionals, which could explain some of the variation we observed in knowledge regarding contraindications to KT. Another methodological limitation is that the data reflected only the views of respondents, who classically tend to express more favorable attitudes than non-respondents. Consequently, the findings that were less expected should be considered even more significant. Of note, this was a pioneering study that may serve as a foundation for improving the methodology of future research. As mentioned previously, this has already occurred.

We identified gaps in knowledge regarding KT contraindications and risk perception as key reasons for non-referral for KT; we also found no barriers at the level of dialysis centers or the healthcare system. This study makes a novel contribution by exploring nephrologists’ perspectives on referral for KT evaluation in a Brazilian state. These findings support the implementation of educational strategies and greater collaboration between nephrology services and KT centers to improve referral. By identifying gaps in knowledge and logistical barriers, this study provides a basis for developing more effective strategies to improve access to KT and reduce disparities in CKD care.

## Data Availability

The datasets generated and/or analyzed during the current study are available from the corresponding author upon reasonable request.
