# Supplementary material for: Barriers to kidney transplant referral in the state of Minas Gerais: nephrologists’ perspectives
Source: J Bras Nefrol. 2026 Aug 10;48(3):e20250215. doi: 10.1590/2175-8239-JBN-2025-0215en (PMC13475452; doi:10.1590/2175-8239-JBN-2025-0215en)
Supplement: Translation

# Translation: Barreiras para o encaminhamento para transplante renal no estado de Minas Gerais na percepção dos nefrologistas

**DOI:** 10.1590/2175-8239-JBN-2025-0215pt

**Authors:** Cristianne de Medeiros Nogueira, Luiza Cunha Martins, Helady Sanders-Pinheiro

**Affiliations:** 1 Universidade Federal de Juiz de Fora, Núcleo Interdisciplinar de Estudos e Pesquisas em Nefrologia, Juiz de Fora, MG, Brasil.; 2 Hospital Regional Dr. João Penido, Serviço de Nefrologia, Juiz de Fora, MG, Brasil.; 3 Universidade Federal de Juiz de Fora, Hospital Universitário, Serviço de Transplante Renal, Juiz de Fora, MG, Brasil.

**Keywords:** Transplante de Rim, Barreiras ao Acesso aos Cuidados de Saúde, Sistemas de Manutenção da Vida, Nefrologistas, Pessoal de Saúde, Encaminhamento e Consulta, Listas de Espera

## Abstract

**Introdução:**

Há baixa porcentagem de pacientes na lista de espera para transplante renal (TxR) no Brasil, mesmo em um sistema público de saúde com cobertura integral. Dos fatores que influenciam o encaminhamento, os relacionados ao nefrologista são os menos estudados. Nosso objetivo foi investigar as barreiras ao encaminhamento para avaliação para TxR sob a perspectiva de nefrologistas em Minas Gerais.

**Métodos:**

Estudo transversal com amostra de conveniência de nefrologistas atuantes no estado. Foram coletadas, por questionário eletrônico, as seguintes informações: características demográficas, formação, atuação, conhecimento sobre indicações (6) e contraindicações (12) e perfil de prática. Análises não paramétricas foram realizadas.

**Resultados:**

Os 77 nefrologistas tinham idade mediana de 44,9 anos (IIQ 39–52), 12,5 anos (IIQ 3–23) de atuação e todos tinham pós-graduação. A mediana de respostas esperadas sobre as contraindicações foi 8 (IIQ 6–9) em 12 possíveis, com 66,2% acertando mais de 50% das questões. Não houve associações significativas com este padrão de respostas (< 8). Os nefrologistas referem conhecer o processo e alegam a presença de contraindicações como principal causa para o não encaminhamento. Os centros eram públicos (54,5%) e não acadêmicos (55,8%), 69,2% estavam localizados a < 100 km de um centro de TxR, a maioria (80,4%) oferecia TxR como modalidade de tratamento, somente 14,7% relataram dificuldades com exames pré-TxR e 21–40% dos pacientes estavam listados (31,2%).

**Conclusão:**

Identificamos falhas no conhecimento das contraindicações para TxR e na percepção de risco como causas para o não encaminhamento. Não foram observadas limitações no nível dos centros ou do sistema. Os resultados fundamentam ações de educação e maior integração entre serviços de nefrologia e centros transplantadores para viabilizar o encaminhamento.

## Introdução

A doença renal crônica (DRC) afeta cerca de 10% da população mundial, com incidência crescente devido ao envelhecimento populacional e ao diagnóstico mais frequente^1^. Quando progride para a fase de falência renal, há necessidade de terapias renais substitutivas (TRS), que incluem hemodiálise, diálise peritoneal e transplante renal (TxR)^2^. No Brasil, a prevalência de pacientes em TRS continua aumentando. O Censo Brasileiro de Diálise estima que, em 2023, havia cerca de 157.357 pacientes em diálise^3^. Comparado às terapias dialíticas, o TxR oferece melhor qualidade de vida e sobrevida^4,5^. No Brasil, a população em diálise aumentou 56,7% em dez anos (2013–2023), enquanto, no mesmo período, o número de TxR aumentou apenas 10,6%^3,6^. Além disso, o crescimento do número de transplantes no Brasil desacelerou nos últimos anos: após um aumento de 42% entre 2008 e 2012, houve apenas 9% de crescimento nos últimos seis anos, o que pode ter sofrido influência da pandemia da COVID-19^7^.

Essa estagnação decorre de barreiras complexas, incluindo fatores do sistema de saúde, questões logísticas, características dos pacientes e desafios na atuação da equipe médica. Entre os fatores mais citados estão a baixa notificação de morte encefálica, a elevada taxa de recusa familiar, dificuldades na manutenção de doadores, a redução no número de doadores vivos e a baixa proporção de pacientes inscritos na lista de espera^8,9^.

Embora o Brasil seja uma referência mundial em transplantes, com o Sistema Único de Saúde (SUS) garantindo acesso integral e gratuito, desigualdades importantes ainda persistem. Em 2024, o país realizou 6.297 transplantes renais, atendendo a apenas cerca de 17% da demanda total, já que havia 36.985 pacientes na lista de espera^7^. A estrutura pública cobre todas as etapas — avaliação pré-TxR, cirurgia, seguimento pós-operatório e imunossupressão —, mas o número de transplantes permanece insuficiente frente à necessidade^7^.

Uma das causas subestimadas dessa lacuna é o acesso à lista de espera para o TxR. A elegibilidade para inscrição requer taxa de filtração glomerular (TFG) reduzida (<10–15 mL/min/1,73 m^2^) sem contraindicações clínicas após avaliação por serviço especializado. Contudo, o primeiro passo depende do encaminhamento médico ao centro transplantador^8,9^. Os poucos dados disponíveis apontam que apenas 17,3 a 33,7% dos pacientes sejam encaminhados e que, destes, menos da metade progrida para a fase de avaliação laboratorial^10,11^. Esse processo é influenciado primordialmente pelas condições clínicas do potencial candidato ao TxR. Entretanto, independentemente do estado de saúde do paciente, esse processo pode ser afetado por características do paciente (idade, escolaridade, sexo, condição socioeconômica), pela atuação dos profissionais de saúde (falta de encaminhamento, desconhecimento das indicações, falhas de comunicação) e por fatores do sistema (distância até centros de transplante, escassez de profissionais)^11,12^. Esse passo crítico e precoce para o acesso ao TxR é pouco estudado, especialmente os fatores além das características dos pacientes^9,12,13,14^.

Os poucos estudos nacionais sobre barreiras ao transplante têm focado majoritariamente na porcentagem de pacientes em lista de espera e no posterior acesso ao TxR, uma vez que este dado é mais factível de ser recuperado^15,16,17,18^. Partindo de dados do Registro Brasileiro de Transplantes e do Censo Brasileiro de Diálise dos anos de 2019 a 2023, a percentagem de pacientes inscritos em lista foi de 18 a 20,9%, com aumento proporcional de 34,5%^3,6,19,20,21,22^. No estado de Minas Gerais, no período de 2016–2019, dados do Sistema Nacional de Transplantes (SNT) e do SUS revelaram que 14,8% dos pacientes estavam cadastrados, com grande variação entre as macrorregiões, de 1,0% em Jequitinhonha a 21,4% na região Norte^23^. Entretanto, somente dois estudos avaliaram aspectos específicos relacionados ao encaminhamento para o TxR^24,25^. Os estudos são de regiões do país com realidades socioeconômicas bem distintas: um da região Sul^25^ e outro do Nordeste^24^. Ambos avaliaram, sob a perspectiva dos pacientes, as causas para a não inclusão em lista ou mesmo para a não realização de avaliação pré-TxR. As causas mais relatadas, inclusive em pacientes com condições clínicas para encaminhamento^24^, foram a falta de informação, a recusa e as dificuldades na realização da avaliação pré-TxR^24,25^. Esses achados reforçam que parte das barreiras pode ser abordada com intervenções simples no nível dos profissionais e do centro de diálise.

Entretanto, não há estudos brasileiros sobre as barreiras para o encaminhamento sob a perspectiva do nefrologista, ator central no início deste processo^14,26^. O estado de Minas Gerais é o segundo estado brasileiro em número absoluto de TxR e o sexto ajustado por população^7^. Em 21 centros ativos, realizou 60% da demanda estimada e tinha 3.737 pacientes em lista, 10,1% do total^7^. O estado ainda conta com uma densidade de nefrologistas maior do que a média nacional^27^. Compreender os fatores que influenciam a decisão e o comportamento do médico ao encaminhar o paciente para avaliação de TxR é essencial para desenvolver estratégias voltadas ao aumento do acesso e à redução das desigualdades^9,26^.

Este estudo tem como objetivo investigar as barreiras ao encaminhamento para avaliação de TxR em Minas Gerais, nos níveis do profissional, do centro e do sistema de saúde, segundo a percepção dos médicos nefrologistas, contribuindo para o planejamento de ações mais eficazes e adaptadas à realidade local.

## Métodos

### Tipo de Estudo e Amostra

Este é um estudo transversal. A amostra foi de conveniência, composta por nefrologistas residentes em Minas Gerais e atuantes no manejo de pacientes com DRC em tratamento conservador ou diálise. Os contatos dos médicos foram obtidos por meio dos registros da Sociedade Brasileira de Nefrologia ou das listas de participantes de eventos promovidos pela Sociedade Mineira de Nefrologia até 2023. Os convites para participação foram enviados entre dezembro de 2023 e junho de 2024, via telefone e/ou e-mail, pelo pesquisador responsável. Nesses contatos, os profissionais receberam um formulário virtual com as perguntas do estudo. Para cada profissional, os convites foram enviados pelo menos três vezes. Os nefrologistas foram incluídos no estudo após aceitarem participar e assinarem o Termo de Consentimento Livre e Esclarecido (TCLE), incluído no próprio questionário eletrônico. As oito seções de perguntas não eram de resposta obrigatória, sendo, portanto, passíveis de serem visualizadas no momento da abertura do questionário. Nas questões sobre indicações para TxR, os participantes respondiam ao enunciado por múltipla escolha com escala do tipo *Likert* (Concordo totalmente/Concordo/Não concordo nem discordo/Discordo/Discordo totalmente). Para as contraindicações, solicitamos o posicionamento também de forma aberta, com as seguintes opções: Contraindicação absoluta/Contraindicação relativa/Não sei. Foi feita inicialmente uma aplicação-piloto com cinco participantes, onde encontramos algumas poucas dificuldades de entendimento que foram posteriormente ajustadas. A opção por esta abordagem teve como objetivo permitir uma exploração mais ampla dos conceitos^28,29^. Foram considerados critérios de inclusão: ser nefrologista atuante no estado de Minas Gerais no cuidado de pacientes com DRC, em tratamento conservador ou diálise. Foram excluídos os questionários com mais de 20% de respostas incompletas.

O estudo foi aprovado pelo Comitê de Ética em Pesquisa em Seres Humanos da Universidade Federal de Juiz de Fora (CAAE 69328523.9.0000.5147; Parecer nº 6.090.544). A participação só era possível após leitura e aceite do TCLE, realizado por clique em campo apropriado no formulário eletrônico que precedia o questionário.

### Variáveis

Foram coletadas variáveis relacionadas a fatores que influenciam o encaminhamento para avaliação para TxR, agrupadas em três categorias principais: características dos nefrologistas, dos centros de diálise e do sistema de saúde^12,13^.

No nível dos nefrologistas estudamos as seguintes variáveis^12,13,28,30,31^: demográficas — idade, gênero e raça (brancos ou não brancos); formação profissional — tempo de graduação (anos), tempo de pós-graduação (anos) e tipo de formação (residência médica, especialização isolada ou ambas); atuação profissional — tipo de serviço em relação ao tratamento da DRC (diálise ou tratamento conservador), vínculo trabalhista com o centro de diálise (prestador de serviço, servidor público, proprietário ou celetista); conhecimento sobre TxR^28,29^ — indicações para TxR (lesão renal irreversível, TFG <10 mL/min/1,73 m^2^ para não diabéticos ou <15 mL/min/1,73 m^2^ para diabéticos, menores de 18 anos ou em casos de doadores vivos, elegibilidade clínica e cirúrgica), contraindicações absolutas (06) (neoplasias malignas, insuficiência orgânica sem tratamento, doenças neurológicas degenerativas, vasculopatia grave envolvendo as artérias ilíacas, doenças psiquiátricas mal controladas, uso de substâncias ilícitas) e contraindicações relativas ou temporárias (06) (perda imunológica recente, infecção aguda, úlcera péptica em atividade, doenças sistêmicas ativas, acidente vascular encefálico (AVE) ou infarto agudo do miocárdio (IAM) recente, doença coronariana não tratada); e motivos para não encaminhamento — contraindicações, percepção de risco maior do que o benefício, falta de doadores.

Dos centros de diálise, avaliamos^11,12,13,32,33,34^: tipo de instituição (acadêmica ou não acadêmica) e natureza econômica (pública ou privada; práticas do centro, incluindo oferecimento de TxR como TRS (sim/não), conhecimento do fluxo de encaminhamento (sim/não), número de nefrologistas, percentual de pacientes inscritos em lista de espera (< 20%, 20–40%, > 40%, não sabe) e profissional responsável pelo encaminhamento (médicos, enfermeiros, assistentes sociais, psicólogos ou ausência de responsável).

Em relação ao sistema de saúde, foram coletadas as seguintes variáveis: distância até o centro de TxR (até 10 km, 10–100 km, > 100 km) e percepção de dificuldade na realização dos exames pré-TxR^12,13,35^.

### Análise Estatística

Foi realizada análise descritiva das variáveis, expressas como média, desvio padrão, mediana e intervalo interquartil (IIQ), conforme a natureza dos dados. A normalidade foi verificada pelo teste de *Kolmogorov-Smirnov*. Para avaliação dos fatores associados ao menor conhecimento das contraindicações para o TxR, dividimos a amostra em dois grupos: menor que a mediana e maior ou igual à mediana das respostas esperadas, que foi 8. Para comparações de variáveis contínuas, foram aplicados o teste t de *Student* ou o teste U de *Mann-Whitney*, conforme a distribuição. Variáveis categóricas foram comparadas pelo teste do qui-quadrado ou pelo teste exato de *Fisher*. O nível de significância adotado foi de p < 0,05. Todas as análises foram realizadas no software jamovi (versão 2.6) (*The jamovi project*, 2025 [*Computer Software*]. *Retrieved from* https://www.jamovi.org).

## Resultados

Dos 496 nefrologistas identificados, 13 foram excluídos: 10 estavam aposentados, 2 não atendiam pacientes e 1 não era nefrologista. Dos 482 restantes, obtivemos 78 respostas, porém apenas 77 continham assinatura do TCLE, perfazendo uma taxa de resposta de 16%. Não houve exclusão de participantes por respostas incompletas (Figura 1). A mediana de idade foi de 44,9 anos (39–52), a maioria (54,5%) era de mulheres, e 76,6% se identificaram como brancos. O tempo mediano de formatura na graduação foi de 18 anos (13–28), o tempo desde a conclusão da pós-graduação foi de 13 anos (6–23) e o tempo de atuação como nefrologista foi de 12,5 anos (3–23). Todos fizeram pós-graduação, a maioria (79,2%) por residência médica, e em 93,5% dos casos a pós-graduação foi realizada em serviço transplantador. Todos relataram trabalhar em serviços que oferecem diálise e tratamento conservador da DRC. O vínculo de trabalho com esses centros, em 68,5% dos casos, era de prestação de serviço e somente 6,8% eram proprietários dos centros (Tabela 1).

**Figura 1:** Composição da amostra estudada.

**Tabela 1:** Características demográficas, de formação e de prática dos nefrologistas atuantes no estado de Minas Gerais e dos centros de nefrologia onde trabalham, de acordo com a mediana de respostas esperadas sobre o conhecimento das contraindicações para TxR

Variável	Total (N = 77)	Respostas esperadas sobre as contraindicações para TxR		p
		< 8 (n = 57)	≥ 8 (n = 20)	
Nefrologista				
Idade, anos	44,9 (39–52)	44,0 (30–64)	42,5 (31–60)	0,42
Feminino, N (%)	42 (54,5)	32 (56,1)	10 (50,0)	0,63
Raça, N (%)				0,68
*Brancos*	59 (76,6)	43 (75,4)	16 (80,0)	
*Não brancos*	18 (23,4)	14 (24,6)	04 (20,0)	
Tempo de graduação, anos	18,0 (13–28)	18,0 (5,0–39,0)	16,0 (6,0–31,0)	0,25
Tempo de pós-graduação, anos	13,0 (6,0–2,03)	13,0 (0–3,07)	12,0 (1,0–2,09)	0,36
Tipo de pós-graduação, N (%)	18 (1,03–28,0)			0,59
*Residência médica*	61 (79,2)	46 (80,7)	15 (75,0)	
*Especialização*	16 (20,8)	11 (19,3)	05 (25,0)	
Pós-graduação em centro transplantador	72 (93,5)	53 (93,0)	19 (95,0)	0,75
Tempo de atuação como nefrologista, anos	12,5 (3–23)	13,0 (1,0–36,0)	12,0 (1,0–31,0)	0,38
Relação de trabalho com o centro, N (%)*				0,09
*Prestador de serviço*	59 (68,5)	41 (75,9)	9 (47,4)	
*Servidor público*	14 (19,2)	7 (12,9)	7 (36,8)	
*Proprietário*	05 (6,8)	03 (5,5)	02 (10,5)	
*Celetista*	04 (5,5)	03 (5,5)	01 (5,3)	
Centros e sistema de saúde				
Financiamento da instituição, N (%)				0,44
*Pública*	42 (54,5)	30 (52,6)	12 (60,0)	
*Privada*	32 (41,6)	27 (47,4)	8 (40,0)	
Instituição acadêmica, N (%)	34 (44,2)	24 (42,1)	10 (50,0)	0,53
Número de nefrologistas	6 (4–10)	6,0 (3,0–20,0)	8,0 (1,0–42,0)	0,17
Oferece TxR como TRS, N (%)	62 (80,4)	47 (82,4)	18 (90,0)	0,42
Distância até o centro de TxR**				0,76
Até 10 km	44 (58,6)	32 (57,1)	12/20 (60,0)	
10–100 km	08 (10,6)	5 (8,9)	3 (15,0)	
> 100 km	23 (30,6)	18 (32,1)	5 (25,0)	
Conhece o fluxo encaminhamento, N (%)	64 (82,9)	45 (78,9)	18 (90,0)	0,59
% de pacientes em lista, N (%)				0,36
< 20%	14 (18,2)	10 (22,8)	04 (20,0)	
20–40%	24 (31,2)	15 (26,3)	09 (45,0)	
> 40%	20 (26)	17 (29,8)	03 (15,0)	
Não sabe	19 (24,7)	15 (26,3)	04 (20,0)	

Abreviaturas – IIQ: intervalo interquartil; TRS: terapia renal substitutiva; TxR: transplante renal. Notas – Dados expressos em número absoluto e percentual ou mediana e intervalo interquartil (IIQ). As comparações entre os grupos de número de respostas esperadas nas contraindicações para TxR foram feitas pelos testes de *Mann-Whitney*, qui-quadrado ou *Fisher*.

*4 valores faltantes: 3 no grupo < 8 respostas e 1 no grupo ≥ 8 respostas.

**1 valor faltante no grupo < 8 respostas.

Sobre o conhecimento relacionado ao encaminhamento, quanto às indicações para TxR, o desempenho foi muito bom. Houve mais de 90% de respostas adequadas em 4 das 6 premissas inquiridas: elegibilidade clínica (97,4%), elegibilidade cirúrgica (92,2%), TFG <10 ml/min/1,73 m^2^ em pacientes sem diabetes (97,4%) e TFG <15 ml/min/1,73 m^2^ em pacientes com diabetes (96,1%). Em relação ao critério de lesão renal irreversível, apenas 71,4% concordaram (Figura 2). Houve um percentual importante de respostas não esperadas quanto à classificação das contraindicações absolutas e um percentual ainda maior nas contraindicações relativas (Figura 3A e B). A mediana das respostas esperadas para contraindicações absolutas (0 a 6) foi de 4,0 (3–5), e para as contraindicações relativas (0 a 6) foi igualmente de 4,0 (3-4). Considerando ambos os grupos de contraindicações (0 a 12), a mediana foi de 8,0 (6–9). Avaliando a amostra pela maioria das respostas esperadas (≥ 50%), 66,2% dos participantes (51/77) identificaram corretamente mais de 50% das contraindicações para TxR. Não encontramos associação significativa entre o desempenho em relação ao conhecimento das contraindicações quando agrupadas pelo acerto da mediana das perguntas (> 8 ou < 8). As razões mais comuns referidas para o não encaminhamento foram a presença de contraindicações (90,8%) e a percepção de risco maior do que o benefício (43,4%), e, menos frequente, a dificuldade de acesso ao centro ou aos exames (21%) (Tabela 1).

**Figura 2:** Frequência de respostas esperadas em relação às indicações para TxR.

Abreviaturas – DM, diabetes mellitus; TFG, taxa de filtração glomerular; TxR, transplante renal.

**Figura 3:** Frequência de respostas esperadas em relação às contraindicações para TxR.

Abreviaturas – A, absolutas; B, relativas ou temporárias; AVE, acidente vascular encefálico; IAM: infarto agudo do miocárdio.

Em relação aos centros de nefrologia, 54,5% eram públicos e a maioria (55,8%) era de centros não acadêmicos. O número mediano de nefrologistas por centro era de 6 (4–10). Mais de 80% relataram rotineiramente oferecer TxR como TRS. Um quarto (24,7%) desconhecia a porcentagem de pacientes inscritos no centro, e 31,2% referiram inclusão em 20% a 40% dos pacientes. (Tabela 1).

Já para os fatores do sistema de saúde, sobre a distância até o centro transplantador, 58,6% dos centros estavam localizados até 10 km de distância, isto é, provavelmente na mesma cidade; 10,6% entre 10 e 100 km; e 30,6% a mais de 100 km. Em relação ao fluxo de encaminhamento, 82,9% afirmaram conhecer os procedimentos, os quais eram realizados em 90,9% dos casos por médicos e assistentes sociais. Somente 17,4% relataram dificuldade para a realização da avaliação laboratorial pré-TxR (Tabela 1).

## Discussão

Este estudo explorou as barreiras ao encaminhamento para avaliação de TxR sob a perspectiva dos nefrologistas de Minas Gerais e como esses fatores podem limitar o acesso à lista de espera. Os resultados demonstram que, nesta população de profissionais com formação em serviços transplantadores e ampla atuação em diálise ou pré-diálise, persistem desafios relacionados ao conhecimento do TxR, principalmente em relação às contraindicações. Por outro lado, não foram relatadas dificuldades nos níveis do centro ou do sistema de saúde. Este é o primeiro estudo brasileiro e um dos poucos no mundo a explorar os fatores que interferem nas fases precoces, de encaminhamento e avaliação laboratorial, do processo de inclusão de candidatos na lista de espera para o TxR^11,12,13,28,30,31,33,34,35^.

A amostra foi composta por profissionais experientes, considerando a idade e o tempo de atuação como nefrologista, com boa formação profissional, incluindo pós-graduação realizada em serviços transplantadores, dos quais mais de 40% atuavam em centros acadêmicos. A média de idade e a distribuição por sexo são similares ao perfil dos nefrologistas especialistas brasileiros^27^. Infelizmente, não foi possível comparar com o perfil dos nefrologistas atuantes no estado de Minas Gerais, pela inexistência desses dados. Por outro lado, esse perfil é contrário ao relatado como fator de risco para o não encaminhamento. Tandon et al.^31^ relataram que ter mais de 10 anos de formação de pós-graduação e não ter filiação acadêmica estão associados ao não encaminhamento. Outra característica relevante é que a maioria trabalhava em serviços públicos, portanto sem fins lucrativos, com vínculo de prestador de serviço, em vez de proprietários do serviço, ambos associados a menor risco de barreiras ao encaminhamento^11,33,34^.

Apesar desse perfil especializado, as pontuações atribuídas às respostas sobre contraindicações revelaram espaço para aprimoramento. Houve dificuldades no conhecimento das contraindicações para o TxR, conhecimento primordial nesta fase, em pelo menos um terço dos respondentes. Esses resultados sugerem heterogeneidade no conhecimento prático sobre os critérios de contraindicação, o que pode impactar as decisões de encaminhamento. Estudos internacionais apontam que incertezas ou percepções equivocadas dos profissionais de saúde constituem barreiras relevantes ao encaminhamento^12,13^. Porém, as falhas de conhecimento são relatadas em grupos específicos, por exemplo, entre pacientes negros^30^ e entre aqueles em que se percebe falha de comunicação entre médico e paciente^36^, mas nenhum estudo avaliou o conhecimento das indicações e contraindicações para o TxR. Uma explicação potencial para o desempenho pode ser o fato de que, apesar de haver um documento de referência internacional^28^, em algumas das indicações/contraindicações as evidências não são robustas e, portanto, trata-se de um tema em evolução. Além disso, o consenso nacional, iniciativa oportuna e essencial da Sociedade Brasileira de Nefrologia, foi publicado apenas recentemente^37^. Infelizmente, não conseguimos, com as variáveis do estudo, identificar características demográficas ou formativas dos profissionais associadas ao perfil de respostas não esperadas, com base na mediana das respostas. Esse achado sugere que as lacunas identificadas no conhecimento aplicado sobre contraindicações para o TxR parecem ser disseminadas de forma relativamente homogênea no grupo estudado, independentemente da idade, do tempo de formação ou do tipo de vínculo profissional, e refletem desafios mais complexos relacionados ao conhecimento aplicado, à prática cotidiana e à organização dos serviços.

Por meio das variáveis que exploraram os centros estudados, não encontramos dificuldades no encaminhamento, pelo perfil de prática relatado de oferta do TxR como TRS, conhecimento do processo e designação de profissionais. Barreiras ao encaminhamento são descritas em centros com sobrecarga de trabalho devido ao número reduzido de profissionais^13,32^ e em centros com fins lucrativos^11,33,34^. Da mesma forma, não encontramos entraves relativos ao sistema de saúde, uma vez que quase 70% referiram ter centros de TxR a até 100 km, portanto com tempo potencial de deslocamento de até 3 horas, variável previamente descrita como barreira^35^. Além disso, uma percentagem inferior a 20% relatou dificuldade de acesso à avaliação laboratorial, sugerindo que, em geral, as premissas de acesso do SUS estão atingindo estes pacientes.

As implicações clínicas deste estudo são significativas. Os resultados sugerem que melhorar o acesso ao TxR demanda intervenções direcionadas não apenas aos pacientes, mas também à capacitação contínua dos profissionais de saúde. Programas educativos específicos, padronização de protocolos nos serviços e reforço do trabalho multiprofissional são ações com potencial de produzir melhoras. Como o nosso país tem grande variabilidade de acesso e realização de TxR, a avaliação de quais seriam as barreiras em cada região poderia trazer um retrato para fundamentar ações nacionais. De fato, este estudo serviu de base para uma pesquisa com os mesmos objetivos realizada em âmbito nacional, ainda sem resultados divulgados (comunicação pessoal, coordenadores do estudo Encaminha Brasil, 06 jul. 2025). Outra estratégia relevante seria a elaboração de diretrizes nacionais para encaminhamento para o TxR, como fonte específica e de fácil acesso, contribuindo assim para a formação e funcionando como referência para os profissionais. Felizmente, o Consenso Brasileiro da Sociedade Brasileira de Nefrologia para Encaminhamento para o TxR foi recentemente publicado^37^.

Este estudo apresenta algumas limitações. A amostra foi obtida por conveniência e restrita aos nefrologistas de Minas Gerais, o que pode limitar a generalização dos resultados para outras regiões ou contextos do país. Também não é possível avaliar com rigor a representatividade para Minas Gerais devido à inexistência de dados demográficos dos nefrologistas do estado. Uma questão importante é a falta de evidências sobre o que pode ser considerado indicação ou contraindicação para o TxR^28^, o que pode ter influenciado o perfil do que consideramos conhecimento. Entretanto, para esta pesquisa, havia a necessidade metodológica de definição operacional do que consideraríamos como indicação e contraindicação. Além disso, não investigamos aspectos relacionados à educação continuada ou a treinamentos específicos recentes dos profissionais, o que poderia ajudar a explicar parte da variação observada no conhecimento sobre contraindicações para o TxR. Pela metodologia, temos também o viés de que os dados se referem aos respondentes, em geral mais positivos em relação ao esperado. Entretanto, por esta razão, valorizam-se mais os achados “menos esperados”. É importante, no entanto, salientar que este é um estudo pioneiro e que pode fundamentar melhorias na metodologia para estudos futuros, o que já aconteceu, como referido anteriormente.

Identificamos falhas no conhecimento das contraindicações para TxR e na percepção de risco como causas para o não encaminhamento para o procedimento, e não observamos barreiras no nível dos centros ou do sistema de saúde. O estudo oferece contribuição inédita ao explorar características dos nefrologistas relacionadas ao encaminhamento para avaliação de TxR em um estado brasileiro. Os resultados fundamentam ações de educação e maior integração entre serviços de nefrologia e centros transplantadores para viabilizar o encaminhamento. Ao identificar lacunas no conhecimento e barreiras logísticas, o estudo fornece subsídios para o desenvolvimento de estratégias mais eficazes para ampliar o acesso ao TxR e reduzir desigualdades no cuidado à DRC.

## Disponibilidade de Dados

Os conjuntos de dados gerados e/ou analisados durante o estudo atual estão disponíveis com o autor correspondente, mediante solicitação.
